# Revisiting Anti-Stokes Emission Features of DNA-Stabilized Silver Nanoclusters

**DOI:** 10.1021/acsomega.4c11384

**Published:** 2025-02-19

**Authors:** Mikkel Baldtzer Liisberg, Giacomo Romolini, Vanessa Rück, Cecilia Cerretani, Tom Vosch

**Affiliations:** Nanoscience Center and Department of Chemistry, University of Copenhagen, Universitetsparken 5, Copenhagen 2100, Denmark

## Abstract

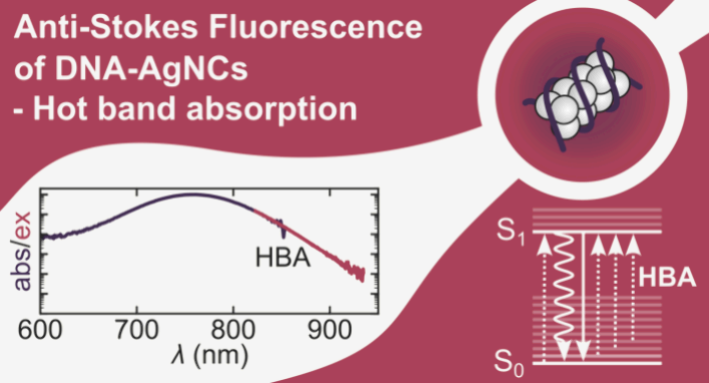

DNA-stabilized silver nanoclusters (DNA-AgNCs) are a class of fluorophores with interesting photophysical properties. They are capable of generating anti-Stokes fluorescence upon excitation with near-infrared lasers. The anti-Stokes fluorescence has previously been speculated to be either the result of consecutive photon absorption (upconversion) or hot band absorption (HBA). Here, we revisit the anti-Stokes fluorescence of DNA-AgNCs to determine the underlying mechanistic origin. We investigate two previously studied DNA-AgNCs together with two organic fluorophores with absorption and emission features in the same spectral regions as the DNA-AgNCs. From the recorded anti-Stokes fluorescence excitation spectra, we find that all the emitters exhibit an exponential-like decaying slope on the red side of the lowest energy absorption band. Furthermore, excitation power dependency measurements at different excitation wavelengths verify the one-photon nature of the anti-Stokes fluorescence. Ultimately, the lack of discrete optical transition features in the anti-Stokes fluorescence excitation spectra suggests that the HBA mechanism is the most plausible cause for the anti-Stokes fluorescence of the investigated DNA-AgNCs.

## 1 Introduction

DNA-stabilized silver nanoclusters (DNA-AgNCs) are intriguing emitters with widely tunable photophysical properties.^1^ Depending on the choice of DNA sequence, different cluster sizes and geometries can be stabilized, which in turn dictate the resulting optical properties.^1^ DNA-AgNCs typically display molecular-like fluorescence characteristics, with absorption and emission features in the visible/NIR and with fluorescence decay times in the nanosecond regime. Similar to molecular fluorophores with triplet states, DNA-AgNCs have been shown to possess long-lived (μs) states.^2^ These states were often labeled as dark, as no appreciable long-lived emission was observed, and were initially probed through fluorescence correlation spectroscopy.^2−4^ However, since the first reports of luminescence from these μs–lived states in 2021,^5,6^ the number of examples has increased steadily.^7−11^ When long-lived emission occurs in conjunction with short-lived (ns) fluorescence, the DNA-AgNCs are classified as dual emitters. There have also been cases where DNA-AgNCs exhibit mainly μs-lived emission, which hints toward the role that the geometry of the AgNC (rod vs spherical) plays in the observed emissive properties.^12^

While the long-lived state was initially labeled as dark, early reports showed that it was possible to utilize the long-lived state for enhancing and modulating the fluorescence signal.^3,13^ This was demonstrated through co-illumination experiments, where a secondary NIR laser (with a wavelength significantly beyond the absorption maximum), in addition to a primary visible excitation source, was used to optically depopulate the long-lived state and increase the overall fluorescence intensity. More recently, it was demonstrated that the depopulation of the long-lived state can lead to a repopulation of the ns-lived fluorescent state, in a process called optically activated delayed fluorescence (OADF).^14,15^ Krause et al. showed that, surprisingly, when using the secondary NIR laser alone, fluorescence could also be observed and that the intensity was linearly dependent on the NIR laser intensity.^15^ An upconversion type model was proposed, where two photons were consecutively absorbed through the microsecond-lived state.^15,16^ The first photon would directly excite the DNA-AgNC from the ground state to the long-lived state, while the second photon would populate the ns-lived state through the OADF process. Hot band absorption (HBA), the process in which vibrationally excited molecules are excited further, was proposed as an alternative model to explain the observed phenomenon. However, this was considered less likely given the large difference in energy between the absorption maximum (573 nm) and the wavelength range of the NIR laser used in the experiments (690–1100 nm).^17^

As similar anti-Stokes fluorescence features have been observed for other DNA-AgNCs solely excited by NIR lasers far away from their absorption maxima, we decided to revisit the mechanistic origin of this peculiar phenomenon. For this purpose, we modified our home-built fluorescence microscopy setup to allow for the collection of accurate anti-Stokes fluorescence excitation spectra and power dependencies.^18^ Here, we present the results on two previously reported DNA-AgNCs and two organic fluorophores that emit in a similar wavelength range, and we demonstrate that HBA is a more plausible model to explain the observed anti-Stokes features. With our findings, we also emphasize the importance of measuring power dependencies at multiple wavelengths when recording two-photon absorption spectra to exclude HBA contributions.

## 2 Results and Discussion

### 2.1 HBA vs UCF

**Figure 1:**
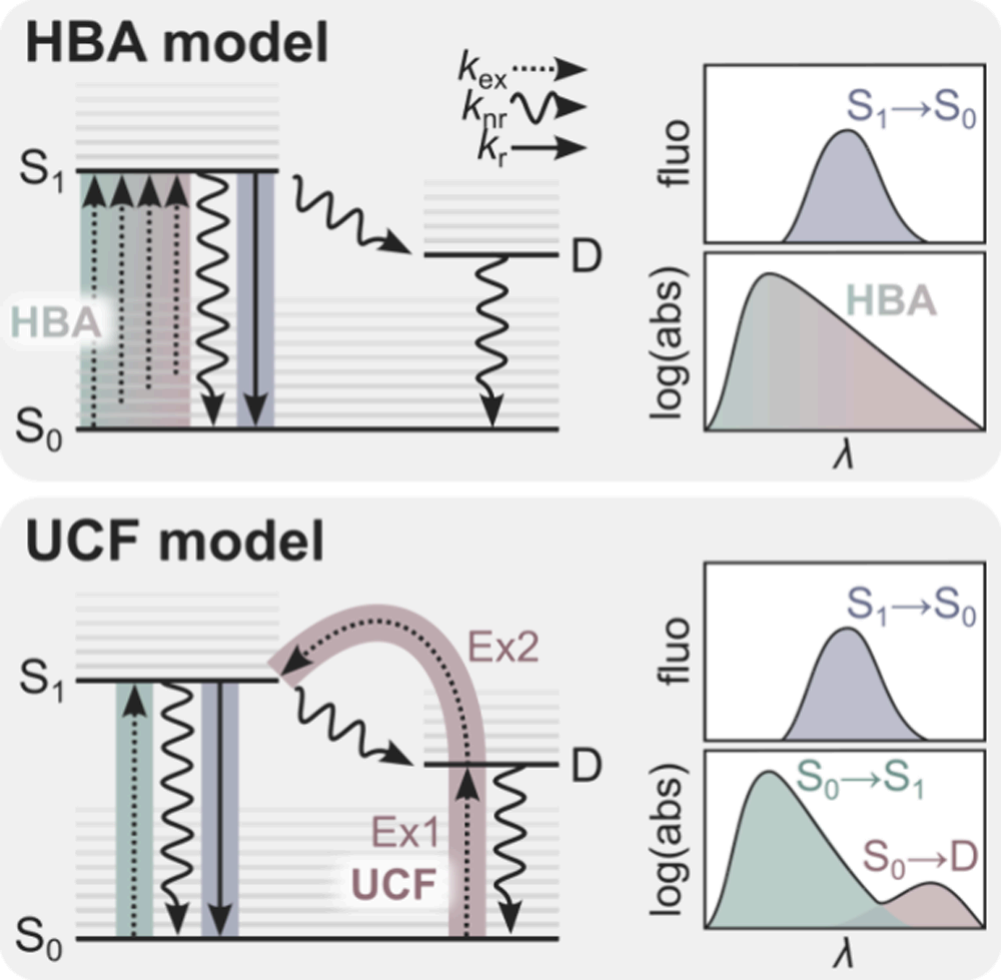
Schematic representation of the two proposed models that could result in the anti-Stokes fluorescence features of DNA-AgNCs. In the HBA model, the anti-Stokes fluorescence features occur as a result of HBA. Here, the fluorescent state (S_1_) is directly excited through a one-photon process from vibrationally excited states of the electronic ground state (S_0_); this gives rise to an absorption feature that is exponentially dropping on the red edge. In the UCF model, the anti-Stokes fluorescence is the result of a two-photon UCF process. For UCF, one photon initially excites (Ex1) the species from S_0_ to the long-lived state (D), followed by the absorption of a second photon that allows for the population of the fluorescent state (Ex2). In the case of UCF, a distinct second absorption band (S_0_ → D) would be expected in addition to the primary absorption band (S_0_ → S_1_). The dotted, wiggly, and solid arrows denote excitation, non-radiative, and radiative rates, respectively. The fluorescence and absorption spectra shown in the figure are cartoons and intended to give an idea of the expected features.

In Figure 1, we show the conceptual differences of anti-Stokes fluorescence generated either by HBA or UCF.^16^ The HBA phenomenon relies on the fact that at a given temperature, the vibrational levels in the ground state (S_0_) are thermally populated following a Boltzmann distribution. This implies that it is possible to directly excite “hot” (vibrationally excited) molecules from the ground state (S_0_) to the excited state (S_1_) with wavelengths significantly longer than the absorption maximum. In other words, some “hot” molecules can still be excited with photons that have less energy than the zero phonon line of the S_0_ → S_1_ transition. In accordance with the Boltzmann distribution, the red edge of the absorption spectrum is expected to drop in an exponential-like fashion, rather than abruptly to zero.

In the UCF case, we assume that the microsecond-lived state can be directly excited from the ground state. Since this transition is significantly less allowed, direct excitation would also have a significantly lower molar absorption coefficient. Nevertheless, as illustrated in the UCF model, some band-like feature related to the direct excitation of the long-lived state should be present in the absorption spectrum. The anti-Stokes fluorescence would then appear after the subsequent absorption of an additional photon to repopulate the fluorescent state, which can be described as an optically activated delayed fluorescence (OADF) step. It is worth noting that, for DNA-640, we have previously excluded that this second step is a thermally activated delayed fluorescence (TADF) process, as the delayed fluorescence was synchronized with the excitation pulse, contrary to what is expected for a thermal process.^19^ Searching for these potential weak transitions in the NIR region via classic absorption spectroscopy is further complicated by the fact that water displays significant absorption features above 700 nm.^20^ While switching to D_2_O could help to alleviate this problem, there is still the uncertainty that any observed features might be due to impurities in the sample. Increasing the concentration of the fluorophore is also an option, but this could lead to aggregation and potential changes in the shape of the absorption spectrum as a function of concentration.

Instead of opting for trying to measure extremely weak absorption features, given the forbidden/less-allowed nature of the S_0_ → D transition, we decided to record fluorescence excitation spectra on the red side of the absorption band using anti-Stokes fluorescence, since this would allow us to verify that the recorded excitation features are indeed related to the fluorophore of interest. We believe that this approach would be the best way to scrutinize the origin of the anti-Stokes fluorescence features of DNA-AgNCs and determine if either the HBA or UCF model best describes our observations. It is worth noting that for HBA, these excitation spectra can be directly related to the absorption spectrum of the fluorophore, while in the case of UCF, the excitation spectra are a convolution of the two absorption processes.

### 2.2 Absorption and Emission Spectra

**Figure 2:**
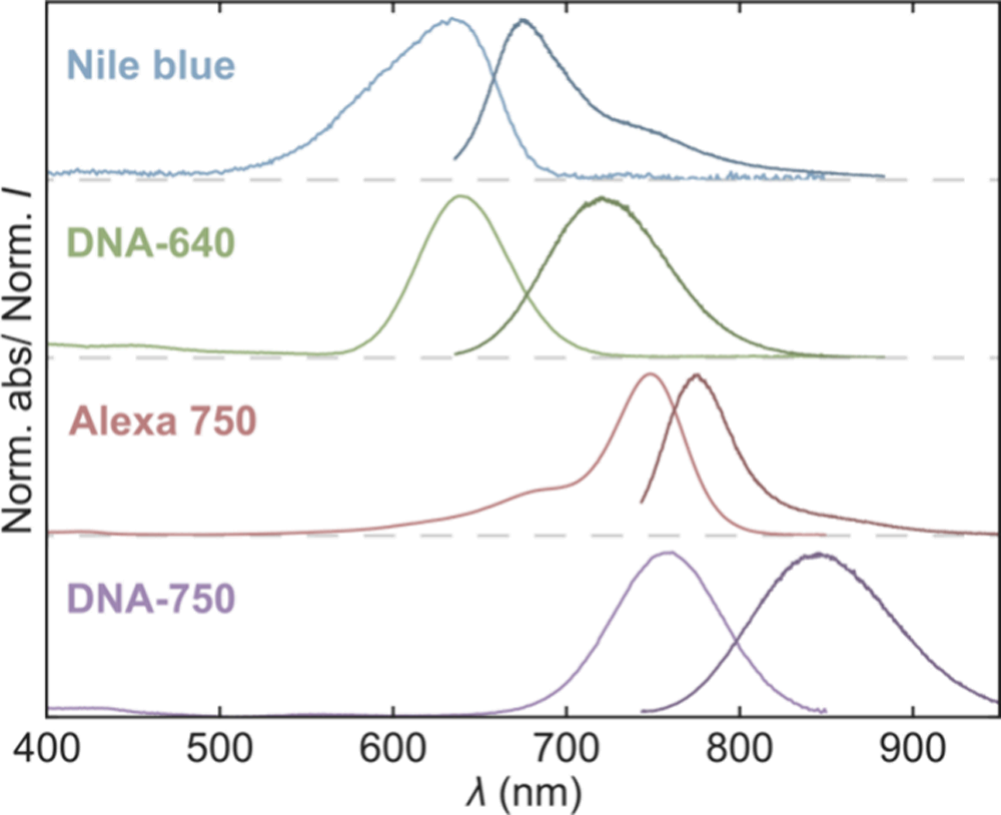
Normalized absorption and emission spectra of the investigated organic dyes (Nile blue and Alexa 750) and DNA-AgNCs (DNA-640 and DNA-750). The emission spectra of Nile blue and DNA-640 were measured with 600 nm excitation, whereas the emission spectra of Alexa 750 and DNA-750 were recorded with 730 nm excitation. All spectra were measured in aqueous solutions. The spectra have been plotted with offsets.

We start by presenting the steady-state absorption and emission spectra of two DNA-AgNCs (DNA-640 and DNA-750) in Figure 2. Details on the synthesis and purification of these DNA-AgNCs are reported elsewhere.^19,21^ We have previously demonstrated that DNA-640 exhibits OADF when co-illuminated by a primary visible excitation beam (640 nm) and a secondary NIR laser (broad excitation from 760–850 nm). However, we also showed that DNA-640 emits anti-Stokes fluorescence when solely excited by the NIR laser, which was classified as UCF at that time.^19^ It has previously been shown by Petty et al. that the fluorescence of DNA-750 could be enhanced upon co-illumination with a secondary 905 nm laser in addition to primary 690 nm excitation, but no significant emission upon sole 905 nm excitation was observed.^3^ To compare our results of the DNA-AgNCs to systems with well-described photophysical properties, we decided to additionally investigate the anti-Stokes fluorescence of two organic fluorophores (Nile blue and Alexa 750) with absorption and emission features in the same spectral range as the two DNA-AgNCs.

From the absorption and emission spectra, a couple of general spectral differences between DNA-AgNCs and organic fluorophores are worth mentioning. Organic fluorophores typically exhibit distinct vibronic structures in both their absorption and emission spectra. Furthermore, they commonly display relatively small Stokes shifts and steep edges on the red side of their low-energy absorption bands. On the contrary, DNA-AgNCs generally show Gaussian-like features in both their absorption and emission spectra. They have relatively large Stokes shifts, and their absorption band extends fairly far into the red owing to its Gaussian-like shape. If one desires to use HBA for anti-Stokes fluorescence imaging, the ideal emitter would have a broad absorption profile with an extended red edge, a small Stokes shift, and a narrow emission feature, as this would enable collecting the highest degree of fluorescence while exciting in a region where the absorption probability remains modest.^22^ In the UCF case, the features of the primary absorption band (at higher energy) would be less important, as the process would proceed through a secondary absorption band (at lower energy) separated from the primary.

### 2.3 Anti-Stokes Fluorescence Excitation Spectra

To record excitation spectra based on anti-Stokes fluorescence, we modified our home-built microscope setup that is normally designed to measure emission spectra in a Stokes configuration (*i*.*e*., the setup we used to measure the emission spectra shown in Figure 2). To construct an excitation spectrum, it is necessary to measure three quantities: the excitation wavelength, the emission intensity of the sample, and the power of the excitation source (to correct for power differences at the different excitation wavelengths). We used a tunable continuous wave Ti:sapphire laser (in the range of 700 to 935 nm) as an excitation source, and an external spectrometer to assess the laser wavelength. Thus, for the purpose of recording excitation spectra, we constructed a setup, where we simultaneously record spectra of the excitation laser (from scatter in the excitation path), emission spectra of the sample, and the power of the excitation laser (on top of the sample). By continuously tuning the excitation laser wavelength and recording the three quantities it is possible to construct excitation spectra based on anti-Stokes fluorescence (see the Supporting Information for details on the setup and data analysis). The optical filters we used for measuring the excitation spectra were chosen based on a balance between collecting sufficient fluorescence and being as close to the absorption maximum as possible (Figure S1).

**Figure 3:**
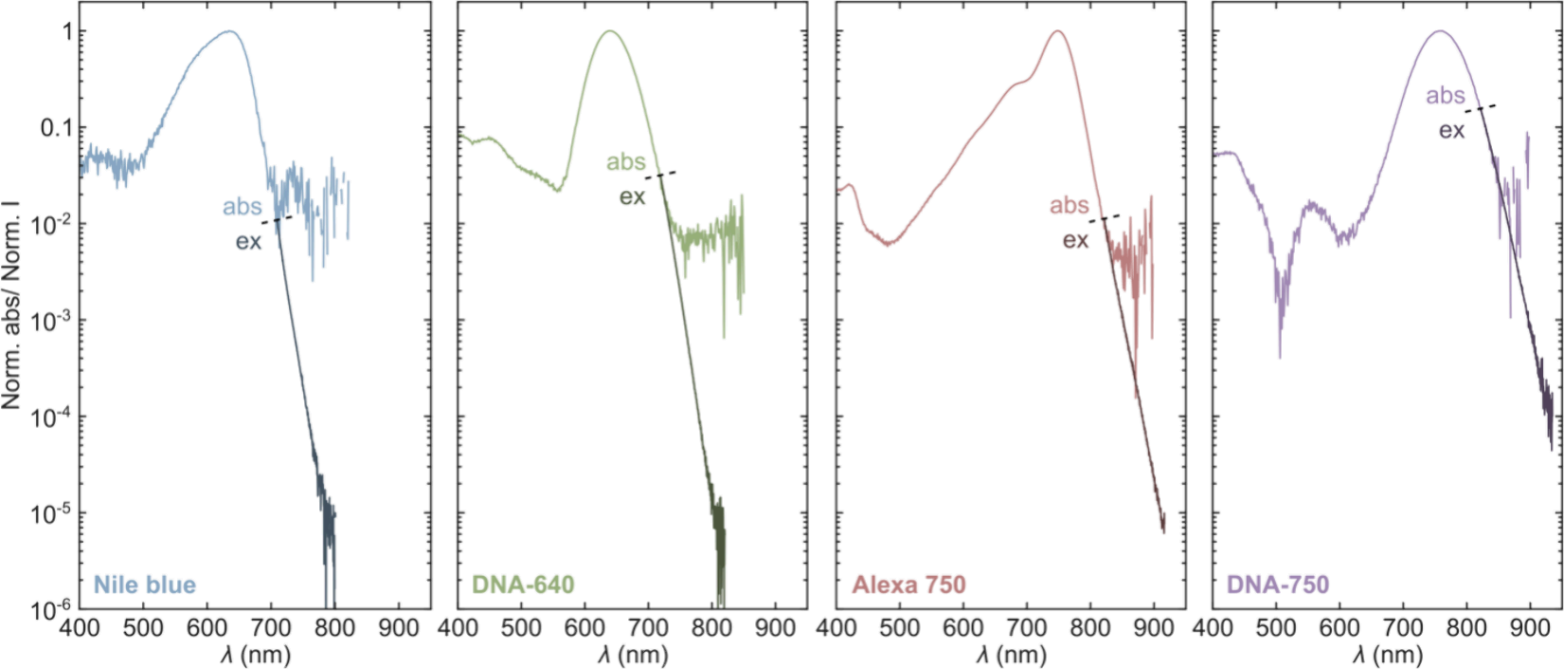
Normalized absorption and scaled anti-Stokes excitation spectra of organic fluorophores (Nile blue and Alexa 750) and DNA-AgNCs (DNA-640 and DNA-750). The excitation spectra are based on anti-Stokes fluorescence, and the beginning of the excitation spectra is marked with a dashed line.

In Figure 3, we show the normalized absorption spectra of the emitters (same spectra as from Figure 2) and the recorded anti-Stokes excitation spectra, which are scaled to match the red tail of the absorption spectra on a log scale. When shown on a log scale, we note that the the noise level of different absorption spectra clearly differs, and a slightly imperfect background subtraction can give rise to rather pronounced features in regions where the absorbance is low (see, for instance, the absorption spectrum of DNA-750 at around 500 nm). While it would generally be preferable to use a higher dye concentration to better assess the red edge of the absorption tail, issues of aggregation effects might alter the absorption features. For example, at higher dye concentrations, Nile blue exhibits a broadening of the red tail and the appearance of a shoulder band, while the spectral features of Alexa 750 remain unaltered and merely display an increased signal-to-noise ratio (Figure S2); accordingly, we show an absorption spectrum of Nile blue in Figure 3 with a low concentration.

For each investigated emitter, the only spectral feature displayed in the excitation spectra is an exponential-like drop toward longer wavelengths. From our combined absorption/excitation spectra measurements, we are effectively able to cover 5 orders of magnitude. With no indication of a second absorption band at longer wavelengths and with the continuous exponential-like drop, the HBA model, rather than the UCF model, seems to better describe the anti-Stokes fluorescence features observed for these two DNA-AgNCs. We are especially confident in this assignment given the comparative measurements with the two organic fluorophores, whose absorption and emission features occur in similar spectral regions. Furthermore, the photophysical properties of organic fluorophores are generally better understood than those of DNA-AgNCs (it is, for instance, still unsettled if the long-lived state of DNA-AgNCs has a triplet character or not)^2,6,23,24^ and HBA has already been established as an explanation for observed anti-Stokes fluorescence features of organic fluorophores.^22,25,26^ It is worth noting that the Boltzmann distribution dictates an exponentially dropping population with increasing energy difference and that our excitation spectra are plotted as a function of wavelength, which is inversely proportional to energy. However, plotting them on an energy scale gives similar-looking features (Figure S3). As we are recording an emission spectrum for every excitation wavelength, we can furthermore confirm that the features of the excitation spectra are solely due to the emitters, and not unwanted contaminants, since the emission features remain unaltered at different excitation wavelengths (Figure S4). The HBA model could also be further validated by performing temperature-dependent anti-Stokes fluorescence measurements at a single excitation wavelength on the red edge.^22^ However, we like to point out that DNA-AgNCs have a limited thermal stability range^19^ and their absorption spectra can broaden and/or shift with temperature,^27^ making the deconvolution of the pure HBA effect extremely complicated.

### 2.4 Power Dependencies

**Figure 4:**
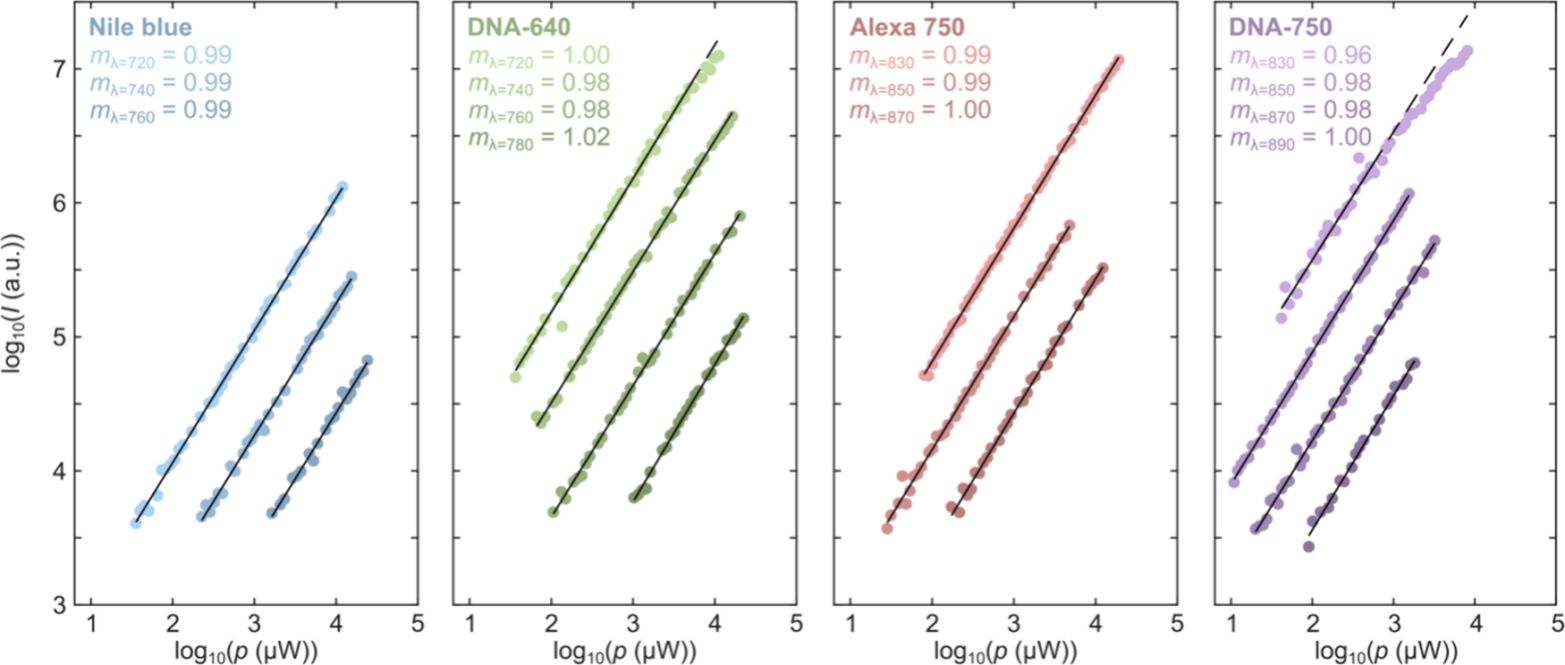
Power dependence of organic fluorophores (Nile blue and Alexa 750) and DNA-AgNCs (DNA-640 and DNA-750) at varying NIR excitation wavelengths. For each of the emitters and excitation wavelengths, the slopes of the linear fits (shown as solid lines) are noted in the legends. Power dependencies of Nile blue were measured at 720, 740, and 760 nm, while DNA-640 was additionally measured at 780 nm. Power dependencies of Alexa 750 were measured at 830, 850, and 870 nm, while DNA-750 was additionally measured at 890 nm. The dashed lines of DNA-640 and DNA-750 at 720 and 830 nm excitation, respectively, show the linear fits extended to the region where the emission starts to saturate, i.e., the range where the data tend to deviate from the employed linear function.

In addition to measuring excitation spectra of the emitters, we also conducted power dependency measurements (see the Supporting Information for details on the experimental setup and data analysis) to assess whether the excitation features we observed were indeed due to one-photon processes. For the HBA model, a linear power dependence is expected, as it entails the absorption of a single photon. The UCF model has a more complicated dependence that is quadratic at low power and linear at higher powers.^17^ For each of the emitters, we checked the power dependence of the anti-Stokes fluorescence at several excitation wavelengths (Figure 4). For almost all of the investigated emitters and excitation wavelengths, we find a power dependence close to 1.00. For the DNA-AgNCs we do, however, see slight indications of saturation at the excitation wavelengths closest to their absorption maxima (λ = 720 nm for DNA-640 and λ = 830 nm for DNA-750) and at the highest powers. This is not unexpected given the significant excitation laser powers used at these conditions (∼10 mW) and the broad Gaussian absorption profile of DNA-AgNCs. From our experiments, we also note a linear spacing between the power dependencies measured at the different excitation wavelengths, which is nicely in line with the exponential behavior observed in the recorded excitation spectra.

Our combined data, with the exponentially dropping excitation features and linear power dependencies, demonstrates that the HBA model fits better than the UCF model for explaining the anti-Stokes features observed for the DNA-AgNCs in these experiments. Besides providing insights on which model best explains the anti-Stokes fluorescence features, we would like to point out the importance of measuring the power dependency when recording excitation spectra; this is especially true when femtosecond lasers are used, where nonlinear (quadratic) dependencies are expected. Here, we showed that it was possible to detect a fluorescence signal even when we excited our samples more than 150 nm above their absorption maximum (Figure 3). Given the nature of HBA characterized by an exponentially decreasing feature on the red edge, increasingly favorable conditions (e.g., higher fluorophore concentration/higher laser intensity/longer integration time) will allow for detecting anti-Stokes fluorescence at increasingly longer wavelengths.

Thus, when recording two-photon excitation spectra^28^ at decreasingly lower excitation wavelengths, which eventually approaches the red tail of the absorption spectrum, assessing the power dependency behavior becomes increasingly important to distinguish HBA from two-photon excitation. Unless the power dependency is measured at each excitation wavelength, assuming two-photon power dependency can lead to errors in the determination of two-photon absorption cross-sections. These considerations are valid for any investigated species that undergo coherent two-photon absorption (TPA).^16^ However, we will solely focus on DNA-AgNCs in the following discussion.

We have previously reported the anti-Stokes fluorescence excitation spectrum (upon femtosecond laser excitation) of a highly studied DNA-AgNC (DNA_2_-[Ag_16_Cl_2_]^8+^),^29−33^ which exhibited a band at around 1045 nm and an exponentially increasing feature toward shorter wavelengths. Fitted power dependencies gave slopes of 1.99 at 1045 nm and 1.76 at 800 nm.^29^ These findings indicate that exciting at 1045 nm (double wavelength of the main 530 nm absorption feature) corresponds to pure TPA, while at 800 nm a combination of TPA and HBA might lead to a subquadratic response. Recently, Hajda et al. reported anti-Stokes fluorescence excitation spectra of four DNA-AgNCs upon femtosecond laser excitation; including DNA-640 and DNA_2_-[Ag_16_Cl_2_]^8+^.^34^ Noteworthy for all four DNA-AgNCs is that their anti-Stokes fluorescence excitation spectra exhibit similar rising tails toward shorter wavelengths. Hajda et al. observed quadratic power dependencies of the DNA-AgNCs far from their increasing tail, and decreasing power dependency values when approaching the tail. For instance, they reported power dependencies of 1.48 ± 0.02 at 810 nm and 1.90 ± 0.01 at 1020 nm for DNA-640. It is not unreasonable that some of the tail features are due to a combination of HBA and TPA and the contribution of HBA keeps increasing when lowering the excitation wavelength. As such, in this wavelength range, the two-photon cross section might be difficult to determine accurately.

## 3 Conclusions

In summary, we have investigated the origin of the anti-Stokes fluorescence features of two DNA-AgNCs. We modified our home-built microscope setup to allow for measuring excitation spectra based on anti-Stokes fluorescence and used it to study two DNA-AgNCs and two organic fluorophores. For all of the investigated emitters, their anti-Stokes excitation spectra showed an exponentially dropping feature toward longer wavelengths, and all their power dependencies showed a linear behavior. These findings, combined with similar observations for organic fluorophores, led us to suggest that the anti-Stokes features of the studied DNA-AgNCs are the result of hot band absorption. This does not exclude UCF as a possible mechanism for other DNA-AgNCs, and the described method represents an ideal approach for testing this. Additionally, with our findings we emphasize that hot band absorption is a general phenomenon for fluorophores, and we note that two-photon absorption cross sections can potentially include one-photon HBA contributions if the power dependence is not carefully checked.

## Supporting Information Available

Details on methods and data analysis, absorption spectra of organic fluorophores at different concentrations, anti-Stokes fluorescence spectra, and excitation spectra on a wavenumber axis (PDF)
