# Supplementary material for: Revisiting Anti-Stokes Emission Features of DNA-Stabilized Silver Nanoclusters
Source: ACS Omega. 2025 Feb 19;10(8):8652–7. doi: 10.1021/acsomega.4c11384 (PMC11886910; doi:10.1021/acsomega.4c11384)
Supplement: Supplementary file 1 — ao4c11384_si_001.pdf [file ao4c11384_si_001.pdf]

Supporting Information

Revisiting Anti-Stokes Emission Features of DNA-Stabilized Silver Nanoclusters.

Mikkel Baldtzer Liisberg,[a,]* Giacomo Romolini,[a] Vanessa Rück,[a] Cecilia Cerretani,[a] and Tom Vosch[a,]*

[a]Nanoscience Center and Department of Chemistry, University of Copenhagen, Universitetsparken 5, 2100 Copenhagen, Denmark.

Email: mbl@chem.ku.dk; tom@chem.ku.dk

1 Materials.

Oligonucleotides and nuclease-free water were purchased from Integrated DNA Technologies (IDT). $AgNO_3$ (≥ 99.998%), $NaBH_4$ (≥ 99.99%) and ammonium acetate (NH_4OAc, ≥ 98%) were purchased from Sigma Aldrich. These chemicals were used as received and dissolved in nuclease-free H_2O. DNA-640 (QY = 73% and Stokes shift = 1791 cm^{-1}) and DNA-750 (QY = 46% and Stokes shift = 1320 cm^{-1}) were synthesized and HPLC purified as described previously.[1, 2]

Nile blue (Stokes shift = 936 cm^{-1}) from Sigma Aldrich and Alexa 750 (Alexa Fluor™ 750 carboxylic acid, tris(triethylammomium) salt; QY = 12% and Stokes shift = 486 cm^{-1})[3] was purchased from Thermo Fisher scientific. Both dyes were used as received and dissolved in Milli-Q water for the measurements.

2 Experimental Setups.

2.1 Absorption Measurements.

The absorption measurements were performed on a Cary 300 UV-Vis spectrophotometer (Agilent Technologies) using a deuterium lamp for ultraviolet radiation and a halogen lamp for visible and near-infrared radiation. The measurements were carried out in a single-beam configuration with a 0/100% transmittance baseline correction. Every spectrum was subtracted by the corresponding blank absorption spectrum.

2.2 Emission Spectra Measurements.

Emission spectra of the emitters were measured on a home-built microscope setup in a Stokes and anti-Stokes configuration. The setups are similar and only differ in the laser excitation source, microscope objective, and optical filters used. The Stokes configuration was used to measure the emission spectra shown in Figure 2, while the anti-Stokes configuration was used for all other measurements of excitation spectra and power dependencies.

2.2.1 Stokes Configuration.

To measure emission spectra in the Stokes configuration, a pulsed 13 MHz fiber coupled (FD7-PM, NKT Photonics) continuum white-light laser (SuperK EXTREME EXB-6, NKT Photonics) was used as an excitation source delivering a wavelength of 600 nm (for Nile blue and DNA-640) or 730 nm (for Alexa 750 and DNA-750) by sending the continuum output through an acousto-

optic tunable filter (SuperK SELECT, NKT Photonics). The output of the fiber was expanded by a telecentric lens system and cleaned up by a band-pass filter ([FF01-600/14-25, Semrock] for λ_{ex} = 600 nm and [TLP01-704-25x36, Semrock] for λ_{ex} = 730 nm). The excitation light was then directed towards the microscope. In the microscope, it was reflected from a 30:70 beam splitter (XF122, Omega Optical) and sent through an oil immersion objective (UPlanSApo 100x, NA = 1.4, Olympus), which focused the laser onto the sample and collected the emission. The excitation light was blocked by a long-pass filter ([LP02-633RE-25, Semrock] for λ_{ex} = 600 nm; no long-pass filter was used for λ_{ex} = 730 nm) and out-of-focus light was removed with a 100 μm pinhole.

The fluorescence was sent through a spectrograph (Acton Research, SP 2356 spectrometer, 300 grooves/mm) onto a nitrogen cooled CCD camera (Princeton Instruments, SPEC-10:100B/LN-eXcelon) for the recording of spectra. The emission spectra were wavelength- and intensity-corrected using calibrated light sources as previously described.[4]

2.2.2 Anti-Stokes Configuration.

For the anti-Stokes configuration, we slightly modified the setup described above. For this, we changed the excitation source to a tunable and more powerful CW Ti:Sapphire laser (Model 3900s, Spectra-Physics) pumped by a solid-state laser (Millennia eV, Spectra-Physics). For cleaning up the excitation path, we inserted a band-pass filter (TLP01-704, Semrock) for Nile blue and DNA-640 and a long-pass filter (HQ815LP, Chroma) for Alexa 750 and DNA-750. To collect fluorescence in an anti-Stokes configuration, we inserted a band-pass filter (TLP01-628, Semrock) for Nile blue and DNA-640 and a short-pass filter (FESH0800, Thorlabs) for Alexa 750 and DNA-750 in the emission path. Note that the wavelengths transmitted by the band-pass filter used for Nile blue and DNA-640 are sensitive to the angle of the filter, which is why there are slight spectral differences between the emission spectra of the two, as the two were measured on different days (see, for instance, Figure S1). We also changed the objective to an air objective (CPlanFLN 10x, NA = 0.3, Olympus). The emission spectra were collected with the same spectrograph/camera, as in the Stokes configuration, but we did not calibrate the spectra, as we were only interested in the relative integrated intensity of the spectra, which is unaffected by this calibration.

2.3 Anti-Stokes Fluorescence Excitation Spectra Measurements.

For measuring anti-Stokes fluorescence excitation spectra, it is necessary to simultaneously record the emission intensity, laser excitation power, and laser excitation wavelength. For this purpose, we constructed a setup (Figure S5) where we simultaneously record all three quantities controlled through self-written LabVIEW software. We used the anti-Stokes configuration described above. To accurately measure the power of the laser illuminating the sample we added a power meter (S120VC, Thorlabs) on top of the sample stage. To assess the laser excitation wavelength, we measured scatter from the Ti:Sapphire laser in the excitation path with a spectrometer (USB2000+, Ocean Optics). Due to software incompatibilities, we measured the emission spectra and laser power on one computer, and the excitation laser scatter on another. For changing the laser wavelength, we utilized a motor controller (SMC100CC, Newport) and associated software (SMC100, Newport) to continuously tune the laser wavelength.

For Nile blue, DNA-640, Alexa 750, and DNA-750 we recorded anti-Stokes excitation spectra in the following ranges: 710-799 nm, 719-820 nm, 822-916 nm, and 821-934 nm, respectively.

2.4 Power Dependency Measurements.

For measuring power dependencies of the emitters, we used the same setup as used for recording anti-Stokes fluorescence excitation spectra, but without the simultaneous measurement of excitation wavelength. Thus, for these measurements, we set the excitation wavelength to a desired value (*e.g.*, 720 nm) and measured emission spectra and the laser excitation power simultaneously. For measuring different power values, we inserted a variable neutral density filter (Thorlabs) in the excitation path and tuned it continuously.

3 Data Analysis

All data analysis and plotting were conducted in MATLAB.

3.1 Excitation Spectra.

An overview of the data analysis procedure for preparing excitation spectra is shown in Figure S6 and will be elaborated upon here. Firstly, the data collected with the setup described in **Section 2.3** is imported (Figure S6i). This data includes a 2D matrix of excitation scatter spectra as a function of time (Figure S6i.a), a 2D matrix of emission spectra as a function of time (Figure S6i.b), and

finally the measured laser excitation power as a function of time (Figure S6i.c). We subtract a background from the excitation scatter spectra and emission spectra (Figure S6i.d).

Secondly, the timescale of the measurements is synchronized (Figure S6ii). As described in **Section 2.3**, we measured the excitation scatter spectra on one computer and the emission spectra and excitation laser power on another. Ultimately, this gives rise to a different number of collected data points. To ensure that the data collected on the different computers is comparable, we blocked/unblocked the excitation laser, which acted as a common temporal marker. Thus, we can clearly identify those data points at which the excitation scatter spectrum (Figure S6ii.a) and laser excitation power (Figure S6ii.b) are similar in time. With the blocking/unblocking events identified, we interpolate the emission spectra and excitation laser power data to match the timescale of the excitation scatter spectra.

Then, we identify the wavelength of the excitation laser at each time point (Figure S6iii). This is simply done by finding the maximum value of the excitation laser scatter spectrum and associated wavelength at each time point (Figure S6iii.a). Doing this for each time point results in a curve of the excitation wavelength as a function time (Figure S6iii.b).

As the wavelength of the excitation laser is tuned, it exhibits major power fluctuations, even for sub-nanometer shifts, and it is thus necessary to correct the emission intensity for the power (Figure S6iv). Firstly, the emission intensity is calculated from the emission spectra by summation within a wavelength range that encompasses the fluorescence features (Figure S6iv.a; in the case of DNA-640, a wavelength range of 636 nm to 714 nm is used). Then, the emission intensity is divided by the laser excitation power at each time point (Figure S6iv.b), which yields a power-corrected emission intensity trace as a function of time (Figure S6iv.c). Note that this power correction of the emission intensity is only valid if there is a linear relationship between the two. At sufficiently high laser powers, one might start to see saturation effects, but we ensure that we are well within a linear regime for our measurements (Figure 4).

With the timescales synchronized and the emission intensity power corrected, it is finally possible to prepare the excitation spectrum (Figure S6v). Thus, we identify each unique excitation wavelength and its corresponding point in time. Since we oversample each excitation wavelength with regard to the emission intensity, we calculate the mean and standard deviation of the emission intensity for each excitation wavelength (Figure S6v.a and Figure S6v.b). Figure S6v.a and Figure

S6v.b show the excitation spectrum as an error bar plot, whereas Figure 3 only displays the mean value for the excitation spectra.

3.2 Power Dependency.

An overview of the data analysis procedure for preparing power dependency curves is presented in Figure S7 and will be elaborated upon here. Initially, the data collected with the setup described in **Section 2.4** is imported, which includes a 2D matrix with emission spectra as a function of time (Figure S7a, b) and the excitation laser power as a function of time (Figure S7c). The emission spectra are background subtracted, and the integrated emission intensity is calculated by summation within a wavelength range that encompasses the fluorescence features. The emission intensity is then plotted as a function of the laser excitation power (Figure S7d). To assess the power dependency, the data is prepared on a log-log plot (Figure S7e). Since some power regions are oversampled (*i.e.*, more points are clustered within a small region of power values), the mean emission intensity within equally spaced power values is calculated on a log-log scale. The corresponding data is then fitted with a linear function (Figure S7f).

To capture the large dynamic range during the power dependency measurements, we encountered some issues with the dynamic range and digitizing capabilities (16bit) of the detection setup for DNA-640 and DNA-750 when we excited our samples closest to their absorption maximum (*e.g.*, 720 nm for DNA-640 and 830 nm for DNA-750) and at high powers. As a means to correct for these effects we used the procedure outlined in Figure S8. First, we identified a spectrum from the power dependency measurements where all the data points were below 65535 (16bit), which we used as a reference spectrum. Then, for spectra that showed regions where 65535 was exceeded and the value was reset to zero by the hardware, we would scale the reference spectrum to have a matching tail in an unaffected wavelength range. This scaling was conducted by calculating the ratio between the sum of the two spectra within this unaffected wavelength range. The corrected spectrum was then calculated as the reference spectrum multiplied by the ratio. Note that an alternative solution could also have been to add 65535 to the data points in the affected region.

4 Additional Figures

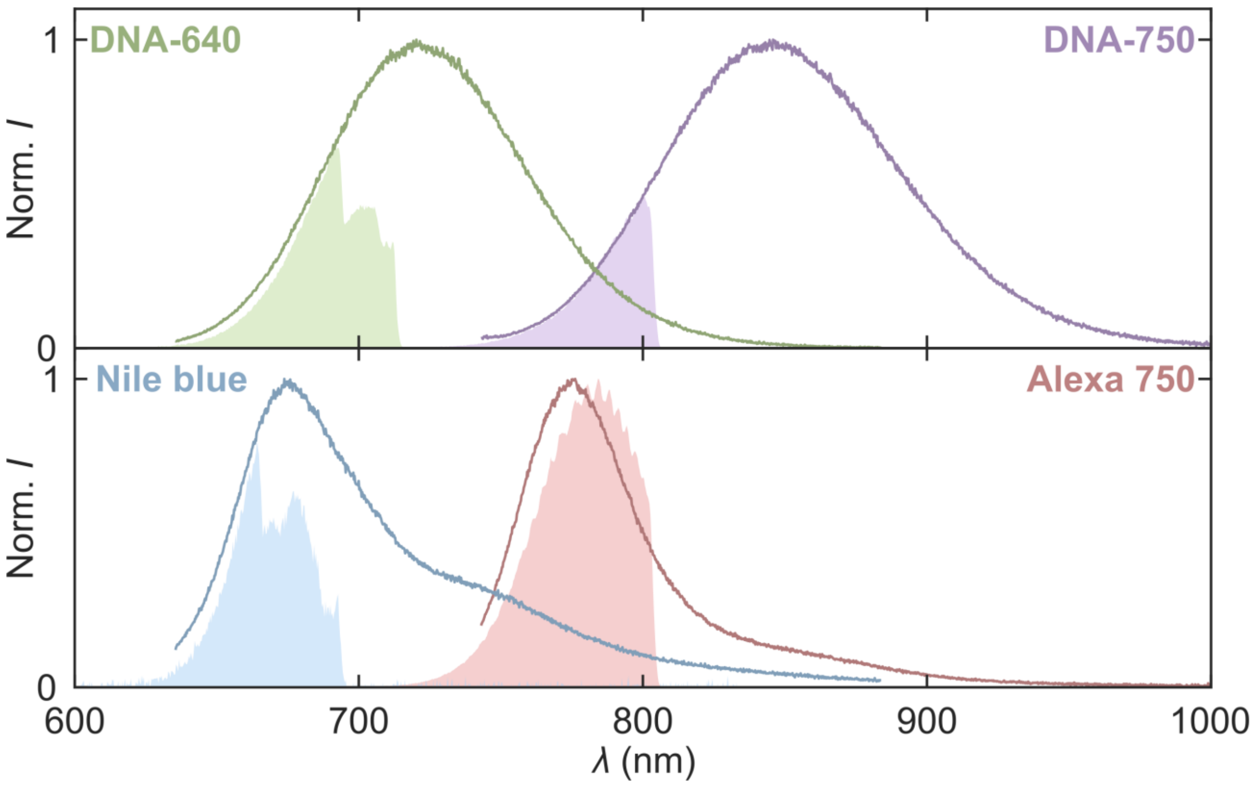

Figure S1: Filtered spectra of the DNA-AgNCs and organic dyes measured in the anti-Stokes configuration (shown as shaded areas) compared with the emission spectra collected under normal Stokes configuration. It must be stressed that the anti-Stokes spectra are not intensity or wavelength calibrated, and that the peculiar features seen for DNA-640 and Nile blue are due to the optical filter used.

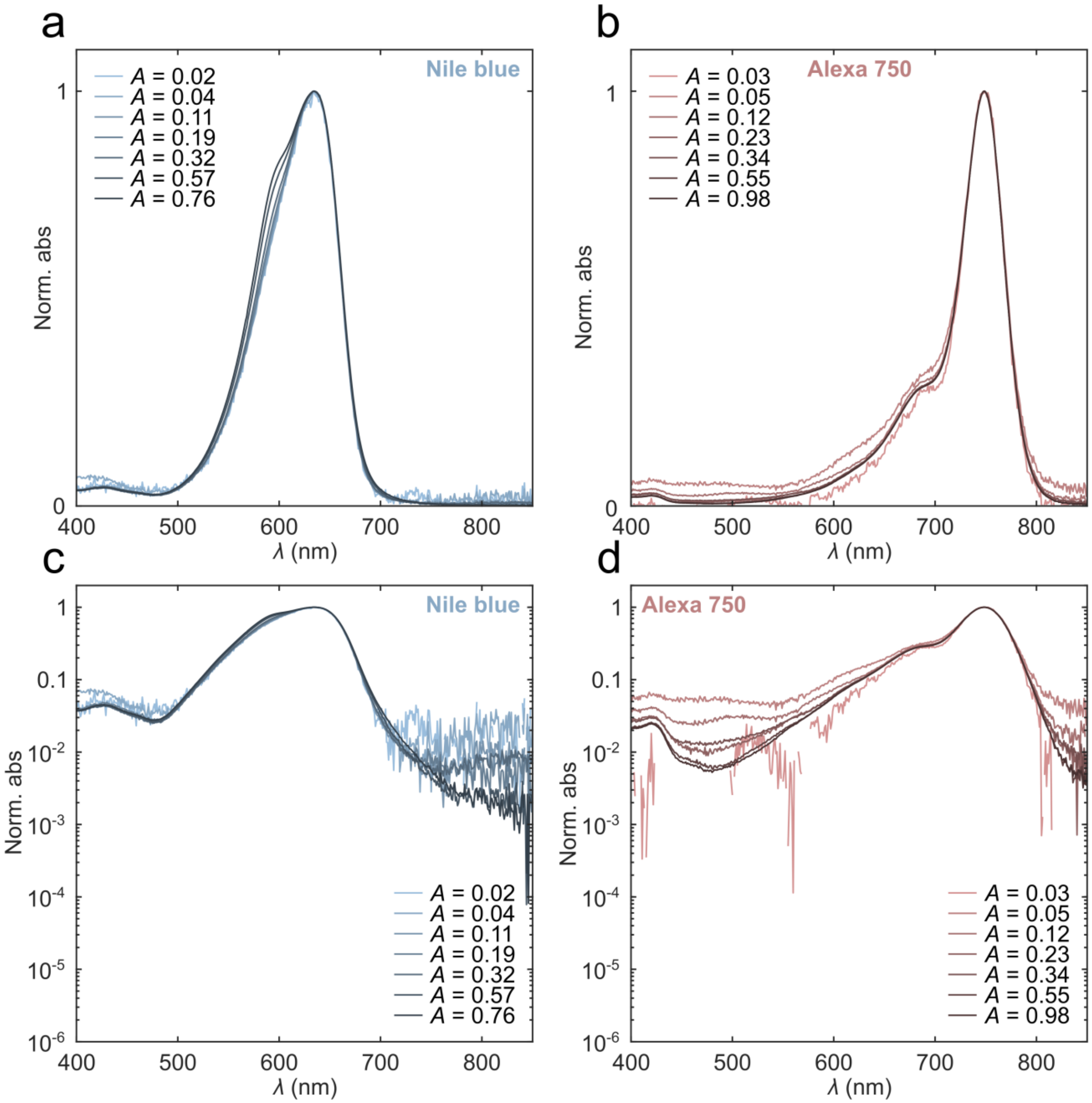

Figure S2: Normalized absorption spectra at varying concentrations of Nile Blue and Alexa 750 dissolved in MQ water shown on a **a, b**. linear and **c, d.** log scale, respectively. Nile Blue shows some spectral alterations at higher concentrations, while Alexa 750 remains unaltered and displays an ever decreasing relative background level.

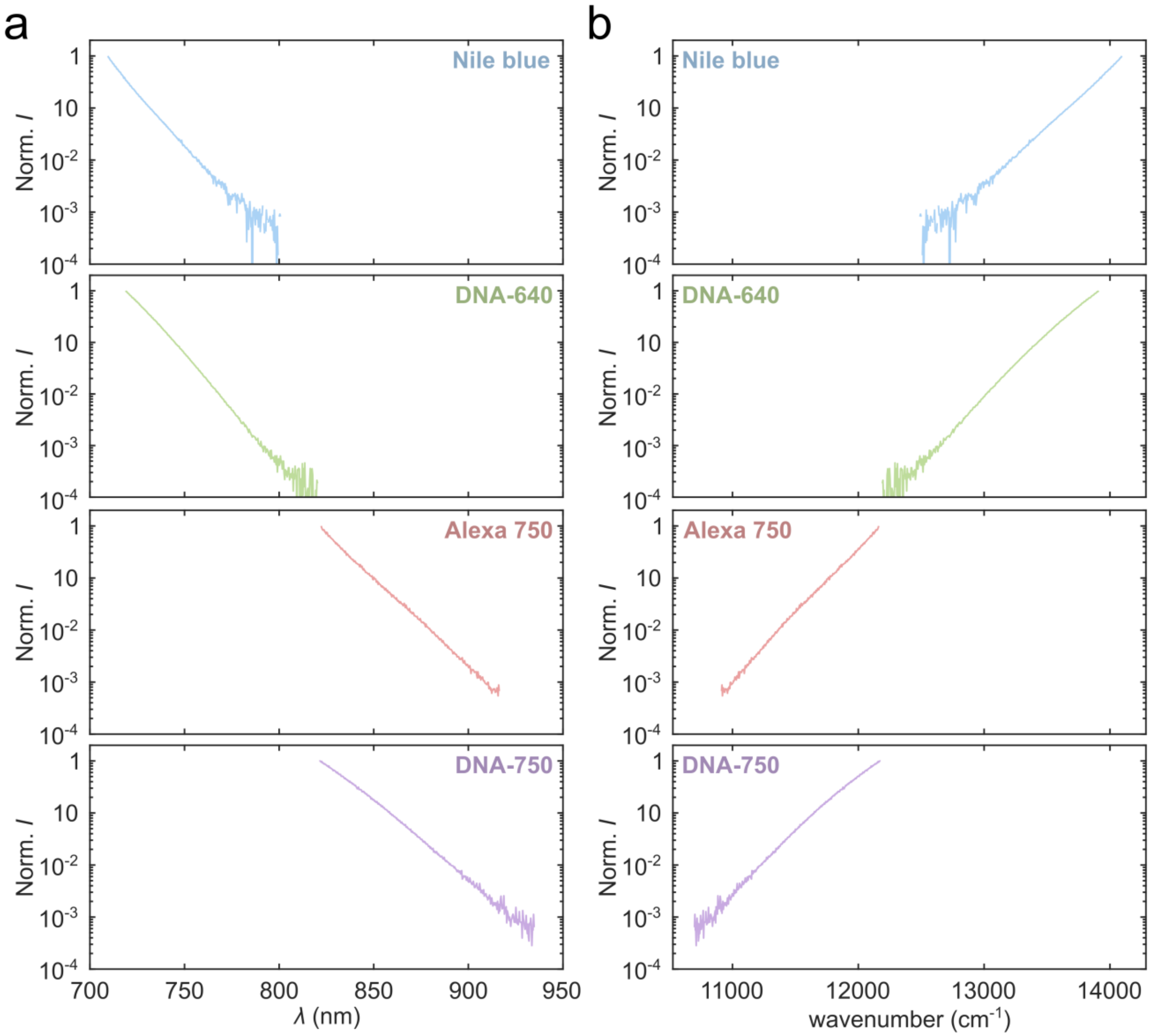

Figure S3: Comparison of excitation spectra plotted on a **a.** wavelength and **b.** wavenumber scale.

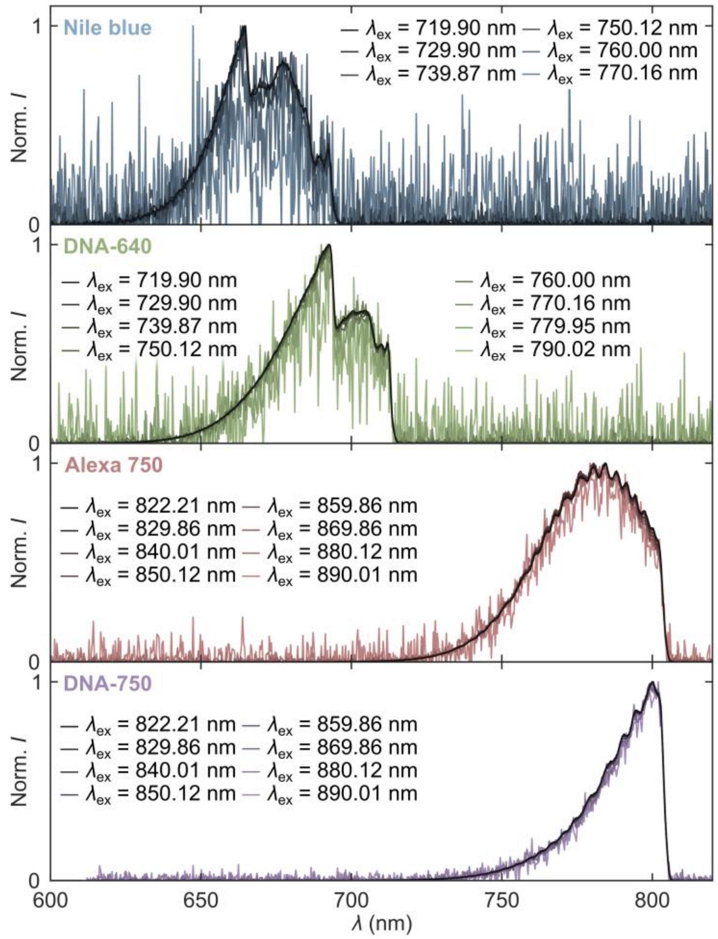

Figure S4: Anti-Stokes emission spectra at different excitation wavelengths. Spectra are represented as the mean of the spectra measured at that particular wavelength, since multiple emission spectra are recorded at each excitation wavelength. The emission features of all emitters remain unaltered at different excitation wavelengths.

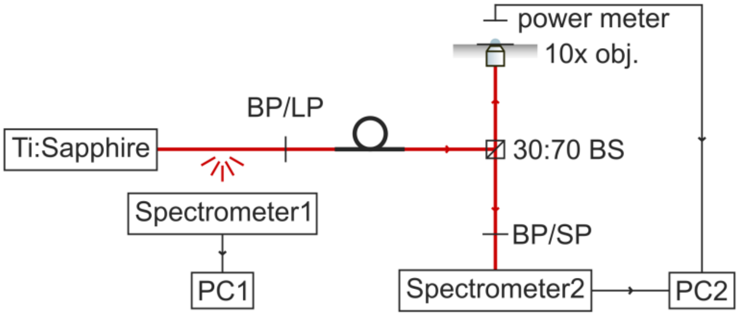

Figure S5: Schematic of the setup used for measuring anti-Stokes fluorescence excitation spectra. Abbreviations used in the figure: BP, band-pass filter; LP, long-pass filter; SP, short-pass filter; BS, beam splitter; obj., objective.

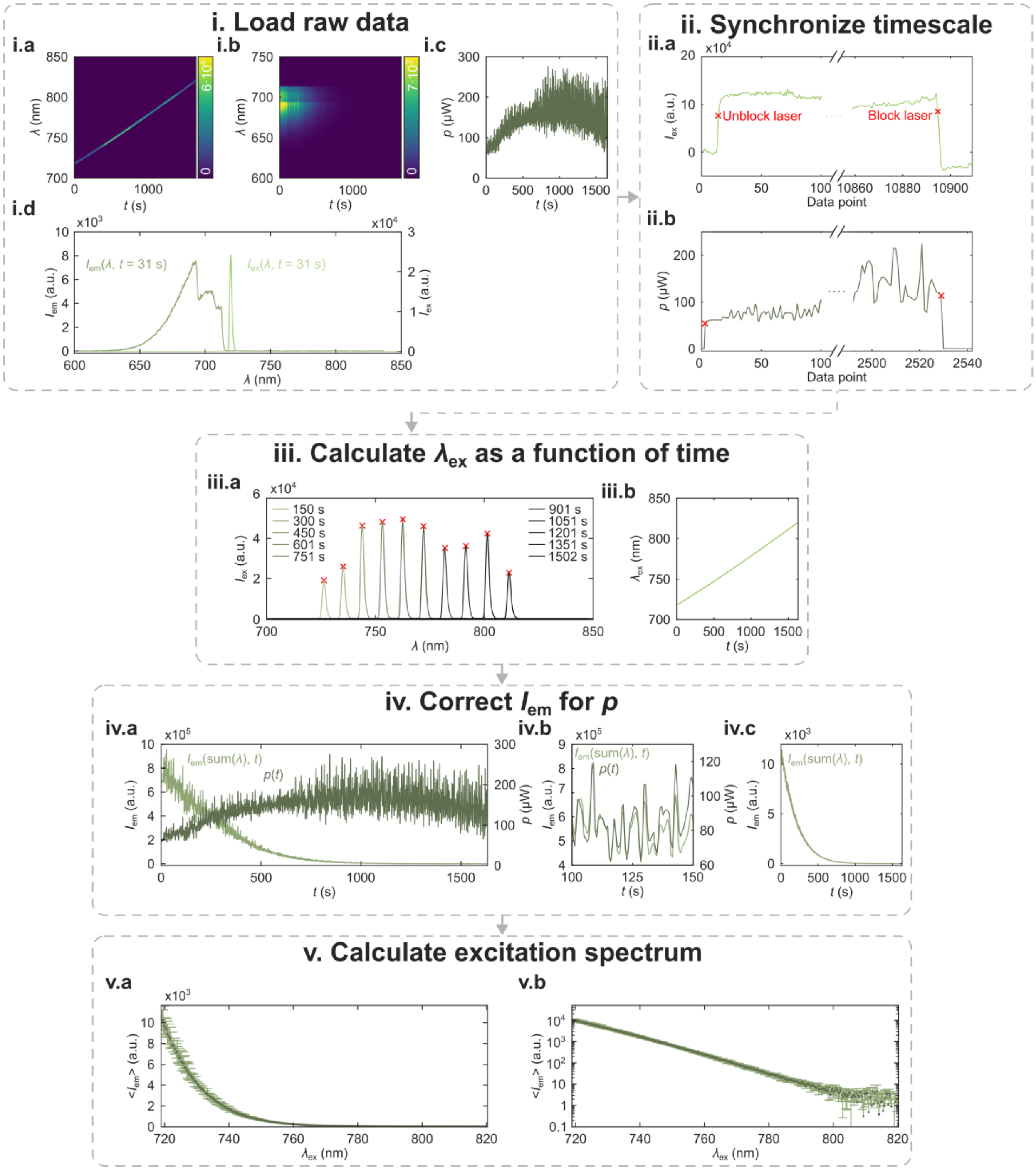

Figure S6: Overview of data analysis framework for the preparation of excitation spectra with data from DNA-640 used as an example. **i.** First, the raw data is loaded, which includes **i.a.** a 2D map of excitation scatter spectra as a function of time, **i.b.** a 2D map of emission spectra as a function of time, and **i.c.** the excitation power as a function of time. **i.d.** An exemplary emission and excitation scatter spectrum at 31 s. **ii.** After loading the data, the timescale of the excitation scatter,

emission, and laser power is synchronized. This is necessary since the excitation scatter is measured on one computer, while the emission spectra and laser power are measured on another computer (different detector deadtimes and integration times also gives rise to different number of collected data points). Unblocking and blocking of the laser is used as a reference (red crosses) for synchronizing the timescale. **iii.** The wavelength of the excitation laser at each time point is calculated simply by locating the maximum value in the excitation scatter spectrum. **iii.a.** Exemplary excitation scatter spectra and located maxima (red crosses), and **iii.b.** the corresponding time resolved wavelength of the excitation source. **iv.** The emission intensity is then corrected for laser power fluctuation as the laser wavelength is tuned by dividing the emission intensity by the laser power at each time point. **iv.a.** Emission intensity and laser power during the whole measurement. **iv.b.** Zoom in of the emission intensity and laser power showing the correlation between emission and laser power fluctuations. **iv.c.** Power-corrected emission intensity as a function of time. **v.** Finally, the excitation spectrum is prepared by calculating the mean of the emission intensity at each identified excitation wavelength. Corresponding excitation spectrum on a **v.a.** log scale, and **v.b.** linear scale, where error bars are the standard deviation.

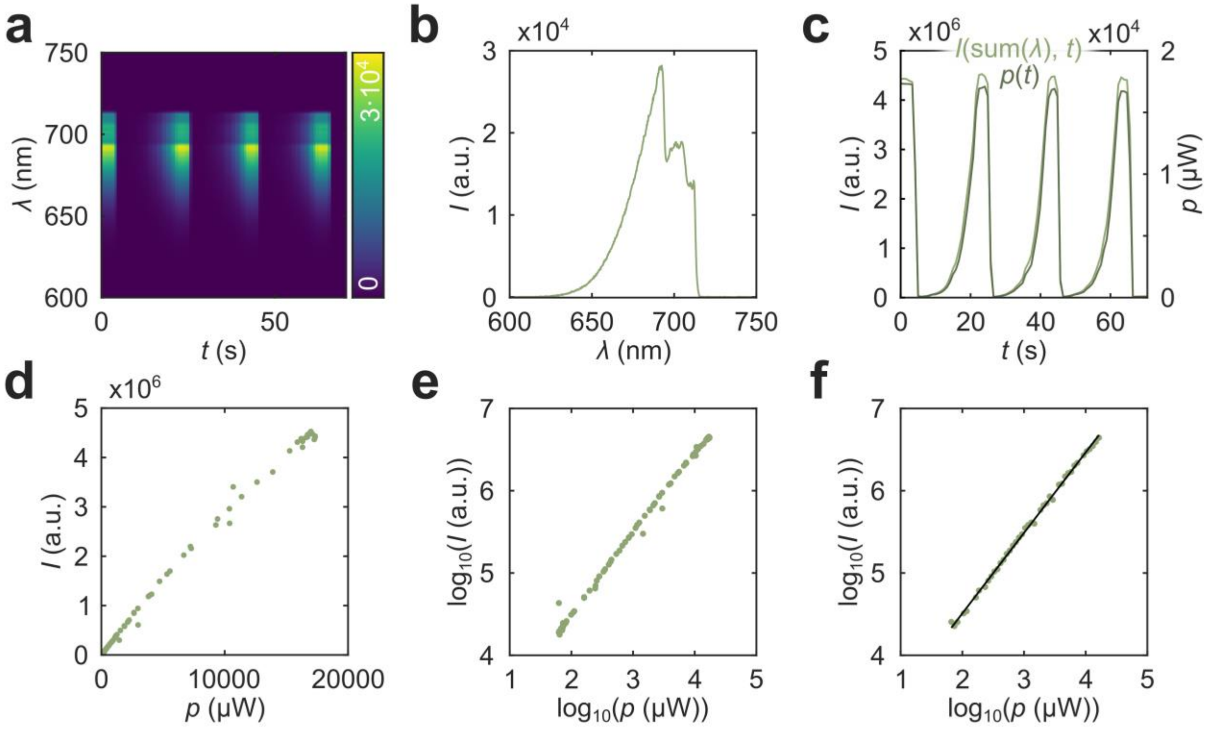

Figure S7: Overview of data analysis framework for the preparation of power dependency curves with data from DNA-640 excited at 740 nm used as an example. **a.** 2D map of emission spectra

collected as the excitation power is changed. **b.** Exemplary emission spectrum (at $t = 0$ s) from the dataset. The summed emission intensity is calculated from the range of 635 nm to 714 nm. **c.** Summed emission intensity and excitation power during the measurement. **d.** Emission intensity as a function of excitation power. **e.** Log-log plot of the emission intensity as a function of the excitation power. **f.** The emission intensity datapoints are binned and fitted with a linear curve.

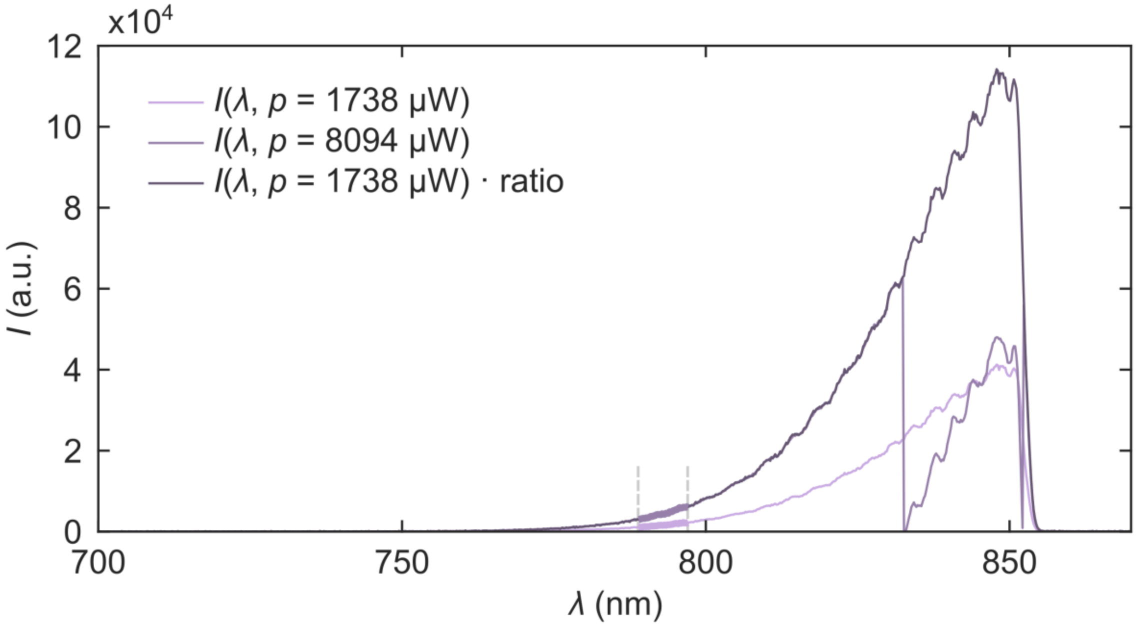

Figure S8: Data analysis approach used to correct for dynamic range issues during power dependency measurements; DNA-750 excited at 830 nm is used as an example. An unaffected reference spectrum ($I(\lambda,\ p = 1738$ μW)) is scaled to match the tail of a spectrum affected by dynamic range issues ($I(\lambda,\ p = 8094$ μW)). The scaling is performed by calculating the ratio between the sum of the two spectra within an unaffected range. Here, a range of 789 nm to 797 nm is used (thick lines bordered by dotted grey lines). The corrected spectrum is then calculated as the reference spectrum multiplied by the ratio.

5 References.

1. Neacşu, V. A.; Cerretani, C.; Liisberg, M. B.; Swasey, S. M.; Gwinn, E. G.; Copp, S. M.; Vosch, T., Unusually large fluorescence quantum yield for a near-infrared emitting DNA-stabilized silver nanocluster. *Chemical Communications* **2020,** *56* (47), 6384-6387.
2. Romolini, G.; Cerretani, C.; Rück, V.; Liisberg, M. B.; Mollerup, C. B.; Vosch, T., Analytical method for the determination of the absorption coefficient of DNA-stabilized silver nanoclusters. *Nanoscale* **2024**.
3. https://www.thermofisher.com/uk/en/home/references/molecular-probes-the-handbook/tables/fluorescence-quantum-yields-and-lifetimes-for-alexa-fluor-dyes.html
4. Liisberg, M. B.; Shakeri Kardar, Z.; Copp, S. M.; Cerretani, C.; Vosch, T., Single-Molecule Detection of DNA-Stabilized Silver Nanoclusters Emitting at the NIR I/II Border. *J. Phys. Chem. Lett.* **2021,** *12* (4), 1150-1154.
